# International Course in Applied Epidemiology

**Published:** 2013-02-15

CDC and the Rollins School of Public Health at Emory University will cosponsor the course, International Course in Applied Epidemiology, from September 23 to October 18, 2013, in Atlanta, Georgia. This basic course in applied epidemiology is directed at public health professionals who work abroad and public health professionals from countries other than the United States. Its content includes presentations and discussions of epidemiologic principles, basic statistical analysis, public health surveillance, field investigations, surveys and sampling, and discussions of the epidemiologic aspects of current major public health problems in global health. Included are small group discussions of epidemiologic case exercises based on field investigations. Participants are encouraged to give a short presentation reviewing some epidemiologic data from their own country.

Computer training using Epi Info 7, a software program developed at CDC for epidemiologists, is included. Prerequisites for the course are familiarity with the vocabulary and principles of basic epidemiology, or completion of CDC’s Principles of Applied Epidemiology home study course, or equivalent. Preference will be given to applicants whose work involves priority public health problems in global health. Registration deadline is June 1 or until filled. Tuition is charged. Additional information and applications are available online (http://www.sph.emory.edu/epicourses); by e-mail (pvaleri@emory.edu); by mail (Emory University, Hubert Department of Global Health, 1518 Clifton Rd. NE, CNR Bldg., Rm. 7038, Atlanta, GA 30322); by telephone (404-727-3485); or by fax (404-727-4590).

